# Electrode Design Based on Porous MnO_2_/PPy Hybrid Nanocomposite and Its Application in Zinc-Ion Batteries

**DOI:** 10.3390/mi16050536

**Published:** 2025-04-29

**Authors:** Shilin Li, Taoyun Zhou, Muzhou Liu, Qiaomei Zhao, Yi Liu, Yun Cheng, Xinyu Li

**Affiliations:** 1 School of Information, Hunan University of Humanities, Science and Technology, Loudi 417099, China; lishilin09@huhst.edu.cn (S.L.);; 2 Key Laboratory of Low-Dimensional Structural Physics and Application, Education Department of Guangxi Zhuang Autonomous Region, College of Physics and Electronic Information Engineering, Guilin University of Technology, Guilin 541004, China

**Keywords:** MnO_2_/PPy hybrid nanocomposite, zinc-ion battery, cycling stability, electrochemical performance

## Abstract

The development of safe, cost-effective, and environmentally friendly energy storage systems has spurred growing interest in aqueous ZIBs. However, the poor cycling stability of cathode materials—mainly due to manganese dissolution and structural degradation—remains a major bottleneck. In this work, a porous MnO_2_/PPy hybrid nanocomposite is successfully synthesized via an in situ co-precipitation strategy. The conductive PPy buffer layer not only alleviates Mn dissolution and buffers volume expansion during cycling but also enhances ion/electron transport and facilitates electrolyte infiltration due to its high surface area. Electrochemical evaluation reveals that the MnO_2_/PPy electrode delivers excellent cycling stability, retaining 75% of its initial capacity after 1000 cycles at a current density of 1 A·g^−1^. Comparative performance analysis shows that MnO_2_/PPy exhibits superior capacity retention and rate capability, especially under high current densities and prolonged cycling. These results underscore the effectiveness of the PPy interfacial layer in improving structural integrity and electrochemical performance, offering a promising route for designing high-performance cathode materials for aqueous ZIBs.

## 1. Introduction

With the gradual depletion of traditional fossil energy sources and worsening environmental pollution, the development of efficient, safe, and environmentally friendly energy storage technologies has become a key focus of global research [1,2,3]. Among various energy storage technologies, secondary batteries play a crucial role in communication, household appliances, and electric transportation due to their high energy conversion efficiency. Therefore, exploring battery systems with high specific capacity, long cycle life, and low cost is essential for advancing energy storage technology [4].

At present, fuel cells and lithium-ion batteries (LIBs) have been widely adopted as mature energy storage technologies in electric vehicles and portable electronic devices. However, the practical application of fuel cells is constrained by challenges in gas storage and slow electrochemical reaction kinetics [5], while the limited availability and high cost of lithium resources have become bottlenecks for the large-scale commercialization of LIBs. Consequently, researchers have turned their attention to sodium (Na) and potassium (K), which are more abundant and cost-effective alternatives [6,7,8]. Despite the advantages of sodium-ion batteries (SIBs) and potassium-ion batteries (PIBs) in terms of resource availability and cost control, the relatively large ionic radii of Na^+^ and K^+^ lead to significant volume changes during the insertion/extraction process, posing considerable challenges in identifying suitable electrode materials [9].

On the other hand, most existing secondary batteries rely on organic electrolytes, which generally present safety risks such as high toxicity, flammability, and explosiveness. In contrast, aqueous electrolytes have gained attention as a safer alternative due to their higher ionic conductivity (approximately equal to 0.1~1 S·cm^−1^, significantly higher than that of the organic electrolytes, which is approximately equal to 10^−3^~10^−2^ S·cm^−1^), excellent rate performance, and lower ohmic polarization characteristics, making them a hot topic in energy storage research [10,11]. Among them, aqueous zinc-ion batteries (ZIBs) have garnered widespread attention due to their abundant zinc resources, high theoretical specific capacity (820 mAh·g^−1^), and environmental friendliness. Additionally, ZIBs can be assembled in an open-air environment without the need for a glove box, simplifying the manufacturing process and reducing production costs, which shows great potential in large-scale energy storage applications [12,13,14,15,16].

Although ZIBs offer many advantages, the cathode materials still face the critical challenge of declining structural stability during long-term charge/discharge cycles, which limits their practical application [17]. The electrochemical performance of electrode materials is closely related to their microstructure, conductivity, and surface properties, all of which are influenced by synthesis methods. Therefore, improving structural stability, electrical conductivity, and charge transfer efficiency through rational material design and optimized fabrication strategies has become a key focus of current research in the energy storage field [18,19]. Moreover, recent studies have shown that device-level engineering—such as functionalized separator design—can also play an essential role in stabilizing cathode materials by regulating ion transport and suppressing active material dissolution, thus contributing to the overall performance enhancement of ZIBs [20].

Recently, MnO_2_ has become an ideal candidate for cathode materials in ZIBs due to its abundant reserves, good reversibility, and variable oxidation states [3]. Studies have shown that nanoscale MnO_2_ can effectively shorten ion diffusion paths and improve electrochemical reaction kinetics by increasing the specific surface area. However, due to the poor inherent conductivity of MnO_2_ and its tendency to dissolve into acidic aqueous electrolytes during cycling, its capacity decays rapidly, severely limiting its practical application. Therefore, optimizing the microstructure of MnO_2_ electrode materials to enhance their conductivity and suppress the dissolution of Mn^2+^ is crucial to improving their electrochemical performance.

In recent years, employing a coating strategy to enhance the cycling stability of electrode materials has become an effective solution. By constructing a protective coating layer, it can not only buffer the volume expansion and contraction of zinc ions during the insertion/extraction process, but can also effectively prevent the dissolution of active materials, thereby improving the cycling life. Researchers have explored methods such as carbon coating, graphene composites, organic conductive polymer coatings, and inorganic compound modifications to optimize the performance of MnO_2_ electrode materials [21,22]. Among them, the conductive polymer polypyrrole (PPy) is considered an ideal coating material due to its excellent flexibility, high conductivity, and superior chemical stability. PPy can not only improve the conductivity of the electrode but also act as a buffer layer to inhibit the dissolution of MnO_2_ during cycling, thus enhancing the structural stability of the electrode [23].

Based on the above research background, this paper employs a simple in situ co-precipitation method to prepare MnO_2_/PPy and systematically investigates its electrochemical performance in ZIBs. Experimental results show that MnO_2_/PPy maintains about 75% of its initial capacity after 1000 cycles at a current density of 1 A·g^−1^ in a 2 M ZnSO_4_ and 0.1 M MnSO_4_ aqueous solution, demonstrating excellent cycling stability. Furthermore, this paper further explores the impact of annealing temperature on morphology, specific surface area, and electrochemical performance of MnO_2_/PPy, providing theoretical support and experimental evidence for the optimization design and practical application of high-performance ZIB cathode materials.

## 2. Material and Methods

### 2.1. Materials

The information on the materials and equipment used in the experiments is shown in Table 1 and Table 2, respectively.

### 2.2. Synthesis of MnO_2_/PPy

In the typical synthesis process, 3.0 g of KMnO_4_ is dissolved in 200 mL of distilled water and stirred continuously for 30 min under magnetic stirring to ensure complete dissolution and the formation of a uniform oxidative environment. Subsequently, 1 mL of pyrrole (PPy) monomer is slowly added dropwise to control the polymerization rate and prevent a violent reaction. As the reaction proceeds, the solution gradually turns brown and a precipitate forms, indicating the successful formation of MnO_2_/PPy.

After the reaction is completed, the resulting precipitate is separated by centrifugation and washed three times each with deionized water and ethanol to remove unreacted precursors and possible by-products, thereby improving the purity of the product. The purified precipitate is then vacuum-dried overnight at 100 °C to ensure complete evaporation of moisture, yielding the preliminarily synthesized MnO_2_/PPy, named MP100.

To further optimize the structure and electrochemical performance of MnO_2_/PPy, thermal treatments at different temperatures are performed. Under an air atmosphere, annealing is carried out at 400 °C and 600 °C for 3 h using a muffle furnace with programmable temperature control to ensure uniform heating, thus enhancing the crystallinity and stability of the material. The resulting samples are designated as MP400 and MP600, respectively. The samples treated at higher temperatures may form more ordered crystal structures, thereby improving their electrical conductivity and ion diffusion properties.

### 2.3. Electrode Preparation and Battery Assembly

Firstly, 70 mg of active material, 20 mg of conductive agent (Super P), and 10 mg of binder (polyvinylidene fluoride, PVDF) are accurately weighed using a precision electronic balance, following a mass ratio of 7:2:1. These materials are successively added to a mortar and thoroughly ground for approximately 20 min to ensure a homogeneous mixture. Subsequently, 15~20 drops of *N*-methyl-2-pyrrolidone (NMP) are gradually added as a solvent while continuously stirring until a uniform slurry is formed.

A stainless steel mesh current collector (diameter: 16 mm, mesh size: 2000 mesh) is used, which is cleaned with anhydrous ethanol prior to use to remove surface impurities. The prepared slurry is then uniformly coated onto one side of the stainless steel mesh and dried for further use (in a separate experimental setup, thermally treated nickel foam with oxygen vacancies is directly used as the anode material without additional preparation and applied directly in battery assembly).

The aqueous zinc-ion battery is assembled as follows [24]:

(1) A CR2016 coin-type cell case is used, and a 16 mm diameter zinc foil (pre-polished) is placed at the bottom as the anode;
(2) The electrolyte used is a mixed solution of 2 M ZnSO_4_ and 0.1 M MnSO_4_;
(3) A Whatman glass fiber membrane (diameter: 19 mm) is employed as the separator;
(4) The assembly sequence is: cell case bottom → cathode electrode → 3 drops of electrolyte → separator → 3 more drops of electrolyte → zinc anode → cell case top cover;
(5) The assembled coin cell is sealed using a coin cell crimping machine to ensure airtightness;
(6) The battery is left to stand at room temperature for 16 h to allow complete infiltration of the electrolyte;
(7) After stabilization, electrochemical performance testing is carried out.

## 3. Results and Discussion

To investigate the zinc storage capability of MnO_2_/PPy as a standalone cathode for aqueous zinc-ion batteries, CR2016 coin cells are assembled according to the method described in Section 2.3 in an ambient environment. The electrochemical performance of the electrode is evaluated using a Neware battery testing system with 2 M ZnSO_4_ and 0.1 M MnSO_4_ as the electrolyte. The cathode performance is assessed through cyclic voltammetry (CV), cycle life testing, and electrochemical impedance spectroscopy (EIS), with the operating voltage window maintained between 0.8 V and 1.9 V.

### 3.1. Characterization of MnO_2_/PPy

#### 3.1.1. Scanning Electron Microscopy (SEM) and Energy Dispersive Spectroscopy (EDS) Analysis

In this study, the scanning electron microscope (SEM) used is the D8 model manufactured by Bruker (Beijing, China) Technology Co., Ltd. During imaging, an electron beam energy of 20 kV is applied. The images are acquired using a secondary electron (SE) detector, which is suitable for observing surface morphology with high resolution.

**Figure 1:**
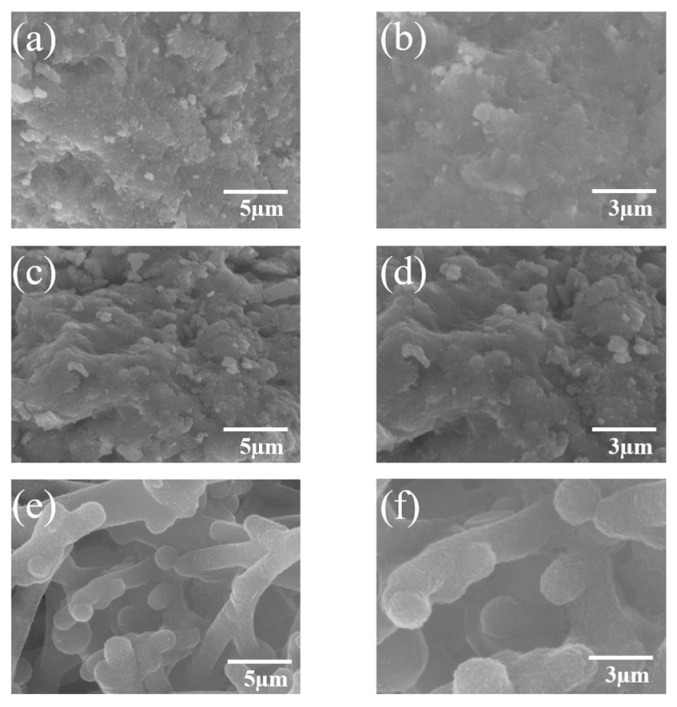
SEM images of MnO_2_/PPy hybrid at different annealing temperatures: (**a**,**b**) MP100, (**c**,**d**) MP400, (**e**,**f**) MP600.

The surface morphology of the material plays a crucial role in its electrochemical performance in aqueous zinc-ion batteries. The SEM images of the prepared samples are shown in Figure 1, providing a microscopic analysis of the morphological evolution of MnO_2_/PPy under different annealing temperatures and their potential impact.

As shown in Figure 1a,b, the MP100 sample exhibits a disordered sponge-like structure with indistinct particle boundaries and no observable crystalline features. This morphology is typically associated with an amorphous or low-crystallinity phase. Due to the lack of well-defined conductive pathways, this structure may restrict ion diffusion and electron transport, thus affecting the overall electrochemical performance.

As the annealing temperature increases, morphological changes become evident. In Figure 1c,d, the MP 400 sample presents a denser texture compared to MP100. Nevertheless, clear crystalline grains are still not discernible in the SEM images. Although thermal treatment at 400 °C may promote partial structural ordering, it remains insufficient to induce a pronounced crystalline morphology observable by SEM.

Upon further increasing the annealing temperature to 600 °C, a significant morphological transformation is observed. Figure 1e,f reveals the formation of a more uniform, worm-like nanostructure composed of interconnected particles smaller than 3 µm. These surface features suggest improved material organization and increased surface area, both beneficial for electrochemical reactions.

It is important to note that SEM imaging provides morphological information but does not allow for a definitive evaluation of crystallinity. Although the structure appears more defined in Figure 1e,f, the degree of crystallinity cannot be accurately determined by SEM alone.

More reliable structural information is obtained through complementary techniques such as X-ray diffraction (XRD) and high-resolution transmission electron microscopy (HRTEM). Therefore, any inference regarding the material’s crystallinity is based on XRD results, as discussed in Section 3.1.2.

**Figure 2:**
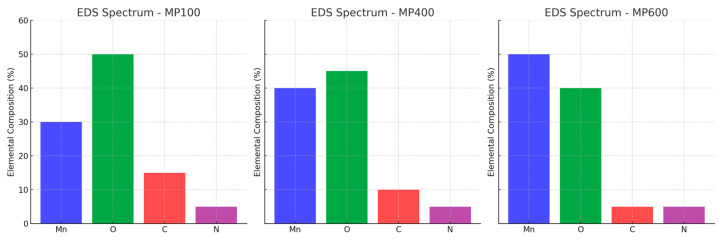
Element distribution of MnO_2_/PPy at different annealing temperatures.

**Figure 3:**
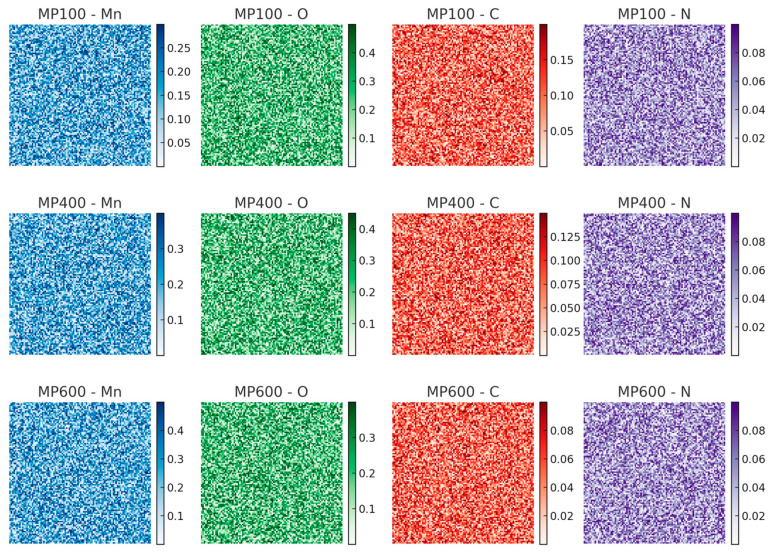
EDS elemental mapping images of Mn, O, C, and N elements for MP100, MP400, and MP600 samples.

To further verify the elemental composition of MnO_2_/PPy composites, energy dispersive spectroscopy (EDS) is performed. The corresponding elemental distribution and mapping images are shown in Figure 2 and Figure 3, respectively.

The EDS spectra confirm the presence of Mn, O, C, and N elements in all samples. Mn and O originate from MnO_2_, and their signal intensities increase with annealing temperature, suggesting structural evolution. C and N derive from the PPy component, whose content decreases at higher temperatures due to partial decomposition. The elemental mapping reveals a homogeneous distribution of Mn and O, while C and N remain well-dispersed, indicating good material integration.

These results support the morphological evolution observed in SEM and corroborate the structural transformation confirmed by XRD analysis.

#### 3.1.2. X-Ray Diffraction Analysis

To further investigate the crystal structure and phase evolution of MnO_2_/PPy composites subjected to different annealing temperatures, XRD analysis is performed using a D8 Advance diffractometer (Bruker, Beijing, China). The measurements are carried out at a tube voltage of 40 kV and a current of 40 mA, employing Cu Kα radiation (λ = 1.5406 Å) as the X-ray source. The diffraction patterns are collected over a 2θ range of 10° to 80°, providing comprehensive insights into the crystallographic characteristics of the samples.

**Figure 4:**
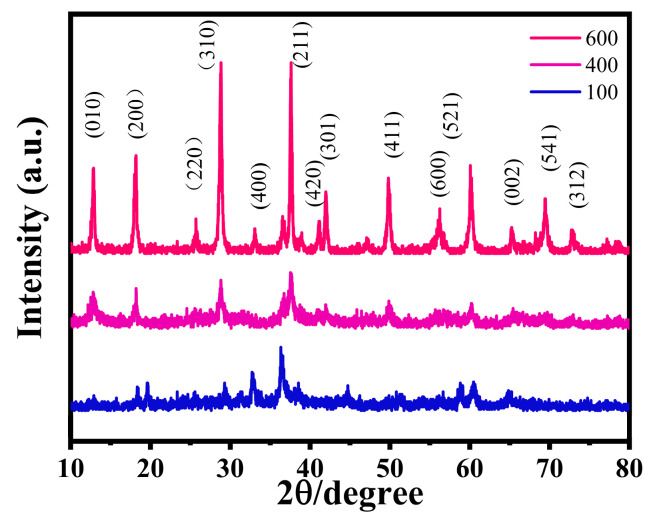
X-ray diffraction (XRD) patterns of the samples at different annealing temperatures.

As shown in Figure 4, significant differences in crystallinity are observed among the samples. The MP100 and MP400 specimens exhibit broad, low-intensity diffraction features, especially around 2θ ≈ 37.5°, which is typically attributed to the (211) reflection of α-MnO_2_ (cryptomelane phase, PDF No. 44-0141). These broad peaks indicate that the MnO_2_ phase is poorly crystalline or even amorphous in the low-temperature samples. This result is in good agreement with the SEM observations in Figure 1a–d, where the corresponding morphologies also appear disordered and lack distinct particle boundaries.

Upon increasing the annealing temperature to 600 °C, the XRD pattern of MP600 reveals several sharp and well-defined peaks. These peaks are indexed to the (110), (200), (310), (211), (301), and (411) planes of highly crystalline α-MnO_2_, confirming that high-temperature treatment promotes a significant enhancement in crystallinity. The emergence of these diffraction peaks reflects the successful formation of a long-range ordered tunnel structure within the MnO_2_ framework.

It is noteworthy that no distinct diffraction signals corresponding to PPy are detected in any of the samples. This absence can be attributed to the partial thermal decomposition of polypyrrole during the annealing process or to its transformation into an amorphous carbonaceous phase lacking long-range order, which is typically undetectable by XRD.

The increased crystallinity at higher temperatures is consistent with the more defined and compact morphological features observed in the SEM images of MP600 Figure 1e,f. The XRD and SEM results together suggest that annealing at elevated temperatures facilitates a structural transition from a disordered to a highly ordered MnO_2_ phase, which is expected to enhance the electron transport, zinc-ion diffusion, and overall structural stability of the composite material.

In summary, the XRD analysis confirms that thermal annealing plays a key role in tuning the crystallinity of MnO_2_/PPy composites. The formation of a well-defined crystalline framework at 600 °C provides structural robustness and improved conductivity, which are beneficial for the material’s application as a high-performance cathode in aqueous zinc-ion batteries.

#### 3.1.3. Thermogravimetric Analysis

The thermogravimetric analysis (TGA) of MnO_2_/PPy is performed using a Netzsch TG 209 F3 instrument (Netzsch-Gerätebau GmbH, Selb, Germany) under an air atmosphere. The temperature is ramped from room temperature to 800 °C at a constant heating rate of 10 °C/min. This setup is selected to simulate the annealing conditions and evaluate the thermal stability and degradation behavior of the PPy component in the composites.

**Figure 5:**
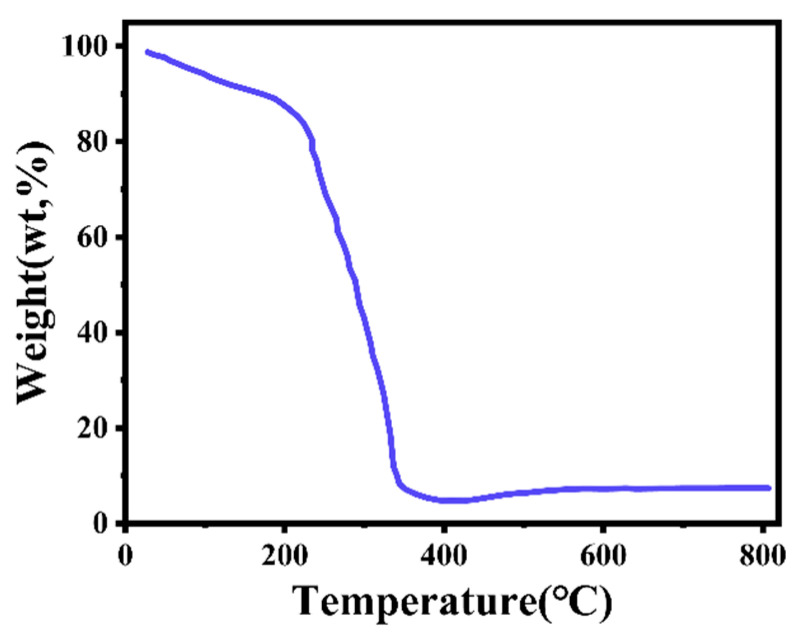
TGA curves of MnO_2_/PPy at different temperatures.

Figure 5 presents the TGA curve of the MnO_2_/PPy in the temperature ranges from room temperature to 800 °C.

The overall weight loss curve can be divided into three distinct stages.

- Stage I (<200 °C):

The sample exhibits a weight loss of approximately 4.8 wt%, which is primarily attributed to the evaporation and removal of physically adsorbed water on the surface or within the pores of the composite. The gradual mass decrease in this stage suggests a relatively low content of adsorbed moisture, indicating that the material possesses a certain degree of environmental stability.

- Stage II (200–350 °C):

A significant weight loss occurs in this temperature range, with a cumulative loss of about 57.4 wt%, representing the most drastic decomposition phase. This pronounced degradation is mainly caused by the release of doped anions (such as Cl^−^ and SO_4_^2−^) and the extensive thermal decomposition of the polypyrrole (PPy) backbone. This stage highlights the relatively low thermal stability of the PPy component within the composite system and represents the dominant phase in the TGA behavior.

- Stage III (>400 °C):

As the temperature continues to rise, the weight loss curve levels off, with only slight changes observed. The residual weight at the end of the TGA test (12 wt%) corresponds to the thermally stable MnO_2_ inorganic component, while the remaining 88 wt% is primarily attributed to the decomposition of polypyrrole (PPy) and other volatile by-products. This indicates that the initial mass ratio between PPy and MnO_2_ in the composite is approximately 88:12.

Additionally, the final residual mass stabilizes at approximately 12 wt%, indicating that the MnO_2_ component retains good thermal stability at elevated temperatures and that most of the organic constituents have been removed.

The TGA results not only confirm the distribution and thermal response characteristics of the organic and inorganic components within the material but also provide valuable insight for optimizing the composite’s structural stability and electrochemical performance under high-temperature conditions.

#### 3.1.4. Nitrogen Adsorption/Desorption Test

**Figure 6:**
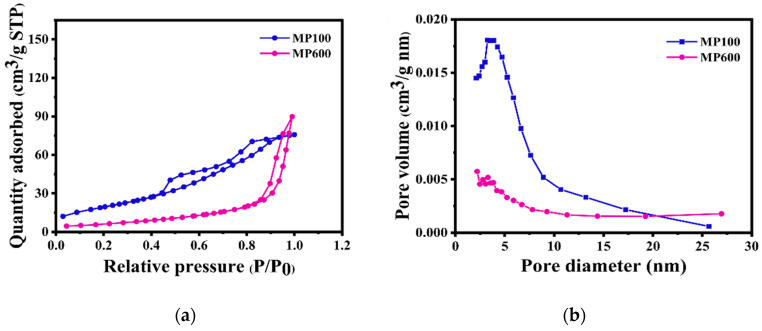
(**a**) Nitrogen adsorption/desorption isotherms of MnO_2_/PPy prepared at different temperatures, (**b**) BJH pore size distribution curves.

Figure 6 presents the nitrogen adsorption/desorption isotherms and corresponding pore size distribution curves of MnO_2_/PPy nanocomposites synthesized at different annealing temperatures, aiming to analyze their specific surface area and pore structure characteristics.

As shown in Figure 6a, both MP100 and MP600 samples exhibit typical type IV isotherms with distinct hysteresis loops observed in the relative pressure range of *p*/*p*_0_ = 0.5~0.99. This indicates the presence of abundant mesoporous structures within the materials, which are beneficial for ion storage and transport. Notably, the MP100 sample shows a significantly higher adsorption capacity compared to MP600, suggesting a larger specific surface area.

Furthermore, the desorption branches of the isotherms are analyzed using the Barrett–Joyner–Halenda (BJH) model, and the resulting pore size distribution curves are shown in Figure 6b. The MP100 sample exhibits a narrow and centered pore size distribution around approximately 6.6 nm, demonstrating well-defined mesoporous characteristics. In contrast, the MP600 sample shows a broader pore size distribution within the 5~20 nm range and a markedly lower pore volume overall. This suggests that high-temperature annealing may have caused partial collapse of the porous framework or decomposition of the organic components, leading to diminished porous features.

According to the BET surface area measurements, MP100 possesses a high specific surface area of 232 m^2^·g^−1^, whereas the surface area of MP600 decreases significantly. This trend of reduced surface area and pore volume is consistent with the results from thermogravimetric analysis (Figure 5) and the morphological evolution observed in SEM images, further confirming that the annealing process at elevated temperatures significantly affects the pore structure of the composites.

### 3.2. Electrochemical Performance

To investigate the zinc storage performance of MnO_2_/PPy as a free-standing cathode for aqueous ZIBs, CR2016 coin cells are assembled in air using MnO_2_/PPy as the cathode, zinc foil as the anode, and a mixed electrolyte of 2 M ZnSO_4_ and 0.1 M MnSO_4_. The electrochemical performance is evaluated using a Neware battery testing system and a CHI 660E electrochemical workstation (CH Instruments, Shanghai, China) at room temperature. The working voltage window is maintained between 0.8~1.9 V.

All electrochemical measurements, including cyclic voltammetry (CV), galvanostatic charge/discharge (GCD), and electrochemical impedance spectroscopy (EIS), are conducted on the CHI 660E system. In the CV tests, the electrode potential is linearly swept at various scan rates ranging from 5 to 100 mV·s^−1^ within the preset potential window. The shape and area of the CV curves are used to assess the capacitive behavior and rate capability of the electrode.

For the EIS measurements, the frequency range is set from 0.01 Hz to 105 Hz with an AC amplitude of 5 mV. The resulting Nyquist plots are fitted using ZView software 4.0 based on appropriate equivalent circuit models. The fitting error is strictly minimized to ensure the accuracy and reliability of the extracted electrochemical parameters. It should be noted that no IR compensation is applied during the measurements.

#### 3.2.1. Cyclic Voltammetry (CV) Test

**Figure 7:**
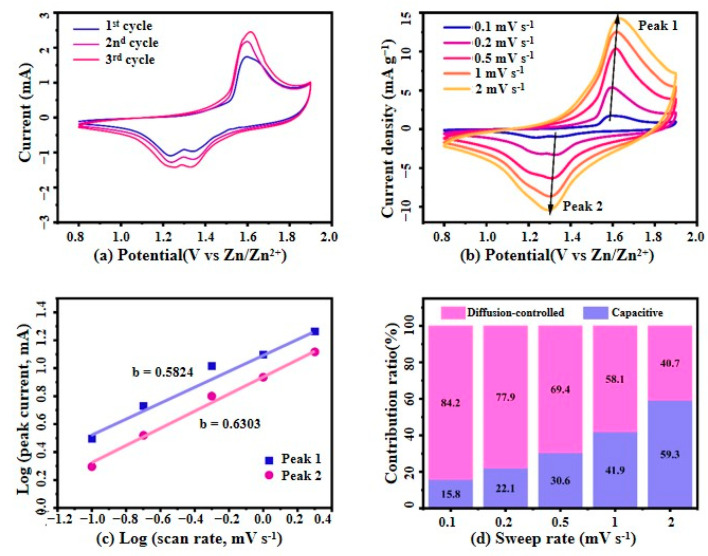
(**a**) The first three CV curves of MnO_2_/PPy at 0.1 mV·s^−1^; (**b**) CV curves at different scan rates; (**c**) Logarithmic relationship between redox peak current (*i*) and scan rate (*v*), along with its correlation with CV data; (**d**) Analysis of the contribution of capacity-controlled and diffusion-controlled processes to the total current at different scan rates.

Figure 7 systematically presents the electrochemical performance of the MnO_2_/PPy electrode, offering an in-depth investigation of its energy storage mechanism through CV curves.

As shown in Figure 7a, the CV curves of the MnO_2_/PPy electrode at a scan rate of 0.1 mV·s^−1^ exhibit distinct redox peaks centered around 1.3 V and 1.6 V. While these peaks were initially attributed to the insertion/extraction of Zn^2+^ into the α-MnO_2_ framework, recent studies and our experimental results suggest a more intricate charge storage mechanism. Specifically, these redox features likely result from a combination of Zn^2+^ and H^+^ co-insertion, reversible Mn^4+^/Mn^2+^ redox transitions, and pseudocapacitive contributions from the polypyrrole (PPy) matrix—particularly under the influence of Mn^2+^ additives in the electrolyte (2 M ZnSO_4_ + 0.1 M MnSO_4_).

The PPy component, accounting for approximately 35% of the composite by mass, as evidenced by EDS and TGA analyses, plays a multifaceted role. Beyond acting as a conductive framework, PPy provides redox-active nitrogen-containing sites that participate in fast surface-controlled reactions, enhancing the pseudocapacitive behavior of the hybrid electrode. Moreover, it facilitates efficient charge transport and mitigates structural degradation during cycling, contributing to the composite’s long-term stability.

The overlapping CV curves during the initial cycles confirm the excellent electrochemical reversibility and structural stability of the MnO_2_/PPy composite. This is attributed to the synergistic effect between MnO_2_ and the PPy buffer layer, which enhances both electronic conductivity and mechanical integrity.

Figure 7b presents the CV curves of the MnO_2_/PPy electrode at various scan rates ranging from 0.1 to 2.0 mV·s^−1^. With increasing scan rates, the peak currents increase correspondingly, and the curves maintain their overall shape, albeit with slightly increased polarization. This behavior suggests a mixed charge storage mechanism involving both diffusion-controlled and surface capacitive processes, which is further supported by the b-value analysis and Dunn’s method discussed in subsequent sections. Together, these results confirm the MnO_2_/PPy electrode’s excellent rate capability, fast charge/transfer kinetics, and stable electrochemical behavior under varying operational conditions.

The relationship between scan rate (*v*) and peak current (*i*) can be described as follows [25]:

(1) $$i=av^{b}$$

The logarithmic form of Equation (1) is:

(2) logi=blogv+log(a)

The calculation of the slope *b* provides a qualitative analysis of the electrochemical kinetics. Typically, the value of *b* ranges between 0.5 and 1.0. When *b* = 0.5, the process is diffusion-controlled, whereas *b* = 1 indicates a surface capacitive process.

Figure 7c presents the log(*i*)–log(*v*) fitting curves for the oxidation peak (Peak 1) and reduction peak (Peak 2). The corresponding *b* values are 0.5824 for Peak 1 and 0.6303 for Peak 2, both falling between 0.5 and 1.0, indicating that the charge storage process of the electrode is governed by a combination of diffusion-controlled and surface capacitive mechanisms. Moreover, the closer the *b* value is to 1, the more dominant the surface capacitive behavior.

According to the semi-empirical method proposed by Dunn et al. [26,27], the total CV current *i* can be divided into two components: the surface capacitive current ($k_{1}v$) and the diffusion-controlled current ($k_{2}\sqrt{v}$), both of which vary with the scan rate. The following Equation (3) is used to characterize and differentiate the contributions of surface capacitive and diffusion-controlled redox processes:

(3) $$i=k_{1}v+k_{2}\sqrt{v}$$

Divide both sides of Equation (3) by $v^{\frac{1}{2}}$:

(4) iv=k1v+k2

where *i* (A) represents the response current, *v* (mV·s^−1^) denotes the scan rate, and *k* is a constant. For a given potential, the surface capacity and diffusion-controlled contribution can be calculated by fitting the linear relationship according to Equation (4).

Equation (4) determines the proportion of surface capacity contribution at different scan rates by utilizing the integral area ratio between the surface capacity response current and the total current.

Figure 7d further quantifies the current components at various scan rates using the Dunn equation, decomposing the total current into diffusion-controlled and surface capacitive contributions. The results show that the capacitive contribution increases significantly with the rise in scan rate, from 15.8% at 0.1 mV·s^−1^ to 59.3% at 2.0 mV·s^−1^. In contrast, the proportion of the diffusion-controlled contribution gradually decreases. This trend indicates that under fast charge/discharge conditions, the surface capacitive behavior plays an increasingly dominant role in the overall current response.

#### 3.2.2. Galvanostatic Charge/Discharge (GCD) Test

**Figure 8:**
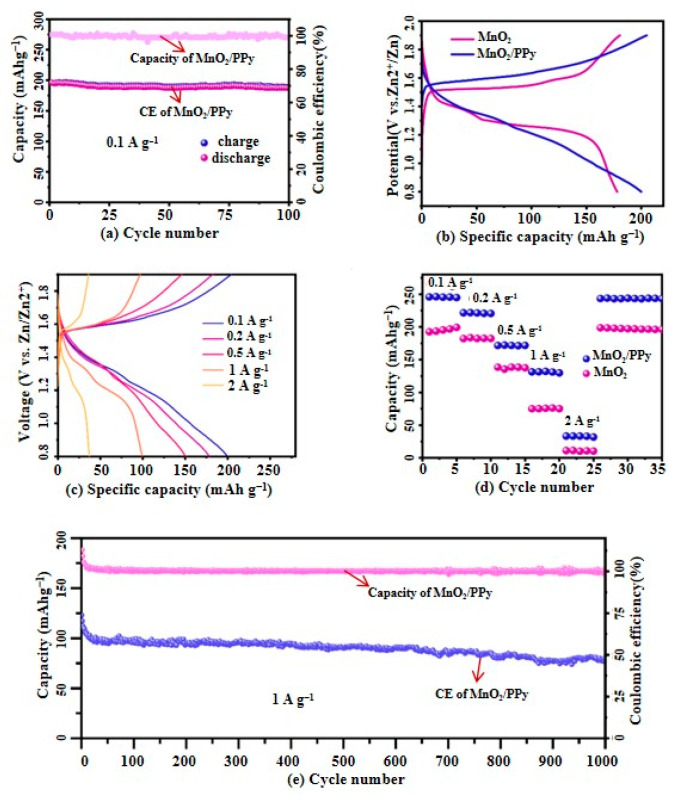
(**a**) Cycling performance of MnO_2_/PPy over the first 100 cycles at a current density of 0.1 A·g^−1^; (**b**) Comparison of charge/discharge curves of MnO_2_/PPy and MnO_2_ at a current density of 0.1 A·g^−1^; (**c**) Charge/discharge performance curves of MnO_2_/PPy at different current densities; (**d**) Rate performance comparison; (**e**) Long-term cycling stability of MnO_2_/PPy at a current density of 1 A·g^−1^.

To evaluate the differences in electrochemical performance among different materials, a series of GCD tests are conducted for comparative analysis, which are shown in Figure 8.

Figure 8a presents the first 100 cycles of the MnO_2_/PPy electrode at a current density of 0.1 A·g^−1^, demonstrating that its capacity remains stable at 205 mAh·g^−1^ with excellent stability. The Coulombic efficiency (CE) remains consistently above 98%, indicating high reversibility and minimal side reactions during charge/discharge processes.

Figure 8b,c compares the charge/discharge curves of MnO_2_/PPy and MnO_2_ electrodes at a current density of 0.1 A·g^−1^, as well as their charge/discharge performance under different current densities.

In Figure 8b, the MnO_2_/PPy electrode shows a higher discharge capacity and lower polarization compared to pure MnO_2_. This demonstrates its enhanced reaction kinetics and improved ion/electron transport capabilities due to the PPy component.

In Figure 8c, when the current density is increased stepwise from 0.1 A·g^−1^ to 0.2 A·g^−1^, 0.5 A·g^−1^, 1 A·g^−1^, and 2 A·g^−1^, the MnO_2_/PPy electrode displays high reversible discharge capacities of 205.2 mAh·g^−1^, 183.2 mAh·g^−1^, 146.3 mAh·g^−1^, 97.3 mAh·g^−1^, and 33 mAh·g^−1^, respectively. At increasing current densities (0.1 → 2 A·g^−1^), the MnO_2_/PPy electrode still exhibits stable and distinguishable charge/discharge plateaus. This demonstrates excellent rate capability, maintaining over 47% of its initial capacity even at a 20× higher current rate.

These findings highlight the outstanding adaptability and performance stability of the MnO_2_/PPy electrode under different load conditions.

Meanwhile, Figure 8d compares the rate performance of the pure MnO_2_ electrode, which shows a relatively lower discharge capacity, further confirming the superiority of the MnO_2_/PPy hybrid nanocomposite. The excellent rate performance of the MnO_2_/PPy electrode suggests that its composite design with polymer nanostructures is both rational and effective.

Figure 8e presents the long-term stability test of the MnO_2_/PPy at a current density of 1 A·g^−1^, demonstrating its exceptional electrochemical performance. Notably, this cathode material initially delivers a high specific capacity of 103.1 mAh·g^−1^ and retains 75% of its initial capacity even after 1000 cycles. Furthermore, its Coulombic efficiency (CE) remains close to 99%, further confirming its potential and reliability for high-performance battery applications.

#### 3.2.3. Electrochemical Impedance Spectroscopy (EIS)

**Figure 9:**
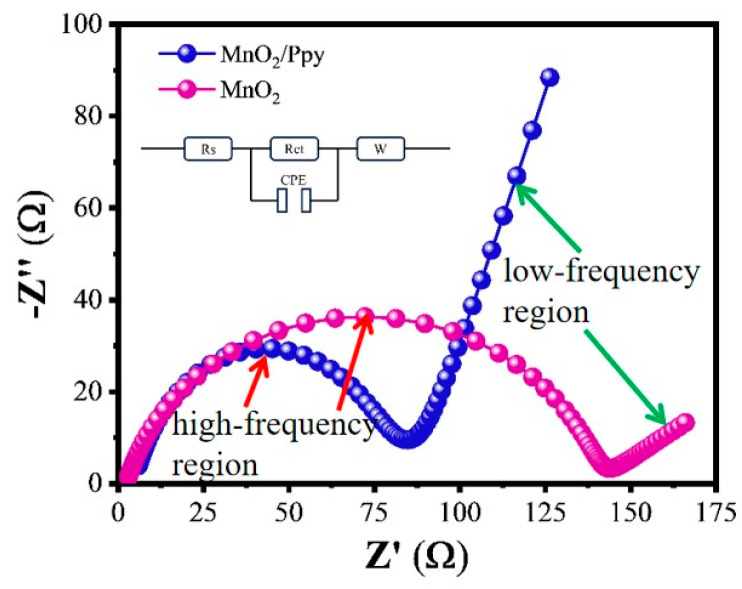
Impedance test of MnO_2_/PPy and MnO_2_.

EIS is an effective technique for characterizing interfacial charge transfer behavior and internal ion diffusion properties of electrode materials. The Nyquist plot shown in Figure 9 consists of a semicircle in the high-frequency region and an inclined straight line at approximately 45° in the low-frequency region at the open-circuit voltage (1.55 V). The high-frequency semicircle mainly reflects the charge transfer process, while the low-frequency line corresponds to the diffusion behavior of Zn^2+^ ions.

Based on the equivalent circuit model illustrated in Figure 9, the charge transfer resistance (Rct) of the MnO_2_/PPy is approximately 87 Ω, which is significantly lower than that of the pure MnO_2_ electrode (~138 Ω). This indicates that the MnO_2_/PPy promotes smoother electronic transport. This enhancement can be primarily attributed to the conductive PPy coating, which effectively improves the overall electrical conductivity of the composite.

Additionally, the MnO_2_/PPy electrode displays a steeper and more linear slope in the low-frequency region of the Nyquist plot, indicating reduced Warburg impedance. This suggests that Zn^2+^ ions encounter less diffusion resistance within the electrode material, resulting in ionic transport. These characteristics confirm the notable advantage of the MnO_2_/PPy composite in facilitating Zn^2+^ diffusion and accelerating electrochemical reaction kinetics.

The updated EIS fitting results (Table 3) further support this conclusion. Compared to pure MnO_2_, MnO_2_/PPy demonstrates significantly lower charge transfer resistance (87 Ω vs. 138 Ω), reduced Warburg impedance (Wo-R and Wo-T), and improved constant phase element (CPE) parameters that are closer to ideal capacitive behavior. Collectively, these results validate that the introduction of PPy enhances both electron and ion transport properties, providing strong theoretical support for the material’s superior rate performance and long-term cycling stability.

### 3.3. Performance Analysis

To comprehensively assess the electrochemical performance of the MnO_2_/PPy hybrid nanocomposite, we conduct a multi-dimensional comparative analysis with several representative MnO_2_-based composite electrodes, focusing on rate capability, cycling stability, and interfacial charge transfer characteristics [3,28,29,30,31]. It is important to note that the literature cited includes both MnO_2_/carbon and MnO_2_/conducting polymer composites, with variations in electrolyte compositions (e.g., pure ZnSO_4_ vs. ZnSO_4_ + MnSO_4_). These differences may influence electrochemical behavior, particularly due to the additional redox buffering and structural stabilization effects of Mn^2+^ ions in mixed electrolytes.

Reference [3] is a prior study from our group that employed MnO_2_ @ C as a baseline for comparison. The electrochemical metrics of all samples are summarized in Table 4, offering qualitative insight into the relative advantages of the MnO_2_/PPy system.

As Table 4 shows, although the electrolyte compositions and composite types vary, the MnO_2_/PPy electrode delivers competitive performance in multiple aspects.

(1) Specific capacity at comparable current densities

While MnO_2_/rGO demonstrates the highest initial capacity (317 mAh·g^−1^ at 0.1 A·g^−1^), its performance under practical long-term cycling or high-rate conditions is less documented. In contrast, MnO_2_/PPy achieves a balanced performance, delivering 205.2 mAh·g^−1^, which is slightly lower than MnO_2_ @ C (210 mAh·g^−1^) and MnO_2_/rGO/PANI (241.1 mAh·g^−1^), but notably higher than traditional α-MnO_2_/super-P systems.

(2) Cycling stability (capacity retention)

MnO_2_ @ C retains 89.5% capacity after 600 cycles at 0.8 A·g^−1^, the highest among all. However, MnO_2_/PPy retains 75% capacity after 1000 cycles at 1 A·g^−1^, showing superior high-rate durability and outperforming α-MnO_2_ and α-MnO_2_/super-P significantly.

(3) Rate performance and high-current adaptability

The MnO_2_/PPy electrode delivers excellent reversibility and stable performance even under fast charge/discharge conditions, retaining over 75% capacity at 1 A·g^−1^. Many reference materials lack high-rate data for comparison, underscoring the robustness of our system.

(4) Electrolyte compatibility

It is acknowledged that electrolytes containing MnSO_4_ (as in our study and ref. [30]) behave differently from pure ZnSO_4_ systems due to Mn^2^⁺’s known role in suppressing MnO_2_ dissolution, buffering pH fluctuations, and participating in redox stabilization. While this introduces some variability, the use of MnSO_4_ is a deliberate design to enhance long-term performance and reproducibility. As such, we clarify that the comparison in Table 4 serves as a contextual benchmark rather than a direct one-to-one equivalence.

In summary, despite differences in composition and electrolyte, the MnO_2_/PPy hybrid electrode demonstrates a favorable balance between specific capacity, rate performance, and long-term cycling stability. These advantages are further supported by impedance data and structural characterization, validating MnO_2_/PPy as a promising candidate among MnO_2_-based aqueous zinc-ion battery cathodes.

## 4. Conclusions

This study presents a simple and controllable strategy to synthesize MnO_2_/PPy hybrid nanocomposites, aiming to address the cycling stability issues of cathode materials in aqueous ZIBs. Systematic investigations reveal that annealing temperature significantly influences the composite’s morphology, surface area, and electrochemical behavior. Electrochemical testing confirms that the MnO_2_/PPy electrode delivers excellent cycling performance, maintaining 75% of its initial capacity after 1000 cycles at 1 A·g^−1^. EIS analysis further highlights its advantages as follows: the charge transfer resistance (Rct) of MnO_2_/PPy is markedly lower (198.7 Ω vs. 368.2 Ω for pure MnO_2_), and its Warburg impedance parameters are also reduced, indicating enhanced ion diffusion and interfacial charge transport. Improved constant phase element values suggest a better capacitive response. Comparative analysis shows that MnO_2_/PPy outperforms many MnO_2_-based electrodes in terms of stability and rate performance. Though its initial capacity is slightly lower than MnO_2_/rGO and MnO_2_/rGO/PANI, MnO_2_/PPy exhibits more robust capacity retention at higher current densities and longer cycles. Its compatibility with neutral electrolytes (2 M ZnSO_4_ + 0.1 M MnSO_4_) further supports its practical application. Taken together, the structural optimization, improved electrochemical kinetics, and synergistic effects between MnO_2_ and PPy make the MnO_2_/PPy composite a promising and practical cathode material for next-generation aqueous zinc-ion batteries.

## Figures and Tables

**Table 1:** Experimental materials.

Material	Purity/Model	Manufacturer
KMnO_4_	AR	Xilong Chemical Corporation (Shantou, China)
C_2_H_5_OH	AR	Xilong Chemical Corporation (Shantou, China)
PPy	AR	MACKLIN Corporation (Shanghai, China)
Zn(SO_4_)_2_	AR	Xilong Chemical Corporation (Shantou, China)
MnSO_4_·H_2_O	AR	MACKLIN Corporation (Shanghai, China)
Fiberglass diaphragm	1823-047	Whatman (a brand of GE Healthcare Life Sciences, now Cytiva, City: Maidstone, UK)
H_2_O	AR	Wahaha Corporation (Hangzhou, China)

**Table 2:** Experimental equipment and instruments.

Equipment	Model	Manufacturer
Analytical Balance	SQP	Sartorius Scientific Instruments (Göttingen, Germany)
Magnetic Stirrer	ZNCL-T	Jinan Sunny Medical Equipment (Jinan, China)
Vacuum Drying Oven	KL-12B	Jingke Instrument Equipment Corporation (Shanghai, China)
Centrifuge	Happy-TL21	Jinan Furuida Machinery Corporation(Jinan, China)
Reaction Kettle	50 mL	Yucheng Apparatus (Shanghai, China)
Cleaner	KQ-600DE	Kunshan Ultrasonic Instruments Corporation (Kunshan, China)
Sealing Machine	MSK-110	Shenzhen Kejing Star Technology Corporation (Shenzhen, China)
Field Emission SEM	MIRA LMS	TESCAN MIRA LMS (Brno, Czech Republic)
High-Resolution TEM	F200X G2	FEI Talos F200X G2 (Hillsboro, OR, USA)
X-ray Diffractometer	D8	Bruker Technology Corporation(Beijing, China)
Raman Spectrometer	alpha300R	WITec alpha300R (Ulm, Germany)
Pore Size Analyzer	ASAP2460	Micromeritics Instrument Corporation (Norcross, GA, USA)
X-ray Photoelectron Spectrometer	K-Alpha	Thermo Scientific K-Alpha (Waltham, MA, USA)
Workstation	CHI760E	Shanghai Chenhua Instruments Corporation (Shanghai, China)
Tube Furnace	OTF-1200X-S	Hefei Kejing Materials Technology Corporation (Hefei, China)

**Table 3:** Analytical data of electrochemical impedance spectroscopy (EIS).

Sample	Rs (Ω)	Rct (Ω)	CPE-T	CPE-P	Wo-R (Ω)	Wo-T (s)	Wo-P
MnO_2_/PPy	5.0	87.2	3.10 × 10^−4^	0.790	78.0	66.0	0.460
MnO_2_	5.7	138.0	2.40 × 10^−4^	0.765	125.0	85.0	0.460

**Table 4:** Comparative analysis of electrochemical performance of selected MnO_2_-based electrode materials.

Materials	Discharge Capacity at Different Current Densities		Cyclic Stability at Different Current Densities and Different Number of Cycles			Electrolyte	Ref.
	Current Density	Discharge Capacity	Current Density	Cycles	Cyclic Stability		
α-MnO_2_/super-P	1 A/g	180 mAh/g	1 A/g	1000	47%	2 M ZnSO_4_	[28]
α-MnO_2_	83 mA/g	233 mAh/g	83 mA/g	50	63%	1 M ZnSO_4_	[29]
MnO_2_/rGO	0.1 A/g	317 mAh/g	2 A/g	2000	78%	2 M ZnSO_4_ + 0.1 M MnSO_4_	[30]
MnO_2_/rGO/PANI	0.1 A/g	241.1 mAh/g	0.1 A/g	600	82.7%	2 M ZnSO_4_	[31]
MnO_2_ @ C	0.1 A/g	210 mAh/g	0.8 A/g	600	89.5%	3 M ZnSO_4_	[3]
MnO_2_/PPy	0.1 A/g	205.2 mAh/g	1 A/g	1000	75%	2 M ZnSO_4_ + 0.1 M MnSO_4_	This work

## Data Availability

The data used to support the findings of this study are included within the article.
